# The comparison of two different plumage-color lines of Japanese quail (*Coturnix japonica*) disclosed a significant effect in increasing abdominal fat contents with increasing age

**DOI:** 10.1007/s11250-023-03601-8

**Published:** 2023-05-02

**Authors:** Abeer A. K. Kirrella, Seham El-Kassas, Saad Mohamed Mostfa, Hassan Hassan Younes, Mostafa Helal, Mohamed Ragab

**Affiliations:** 1 Poultry Production Department, Faculty of Agriculture, Kafrelsheikh University, Kafrelsheikh, Egypt; 2 Animal, Poultry and Fish Breeding and Production, Department of Animal Wealth Development, Faculty of Veterinary Medicine, Kafrelsheikh University, Kafrelsheikh, Egypt; 3 Department of Animal Production, Faculty of Agriculture, Cairo University, Giza, Egypt

**Keywords:** Japanese quail, Plumage color, Carcass traits, Total body fat, Intestinal morphology

## Abstract

This study aimed to investigate the characteristic differences between the white and brown-feathered Japanese quails, by evaluating the carcass traits and egg fat content, blood parameters, and intestinal histopathological features. A total of 1200 1-day-old Japanese quail chicks of two varieties (brown and white-feathered) were used in this study. Live body weight and feed intake were reordered every week. At the 4^th^ week of age, 80 birds from each variety were slaughtered and carcass quality measurements and histopathological changes were recorded. After 6 weeks of age, eggs were collected, and egg quality was assessed. The results revealed that white-feathered quails had significantly heavier body weights and higher growth rates. At 4 weeks of age, females of the white-feather quail had significantly heavier slaughter, after de-feathering, and carcass weights. Remarkable variations between the studied quail varieties, with significant dominance of females in both varieties, at the level of water holding capacity, pH, and meat tenderness ascertained an obvious superiority of white-feathered quails compared to brown ones and indicated the higher tendency of the white quails for meat production. These results were linked with significant changes in biochemical profiles including lipids biomarkers, total protein, and Ca and phosphorus levels along with variations in the intestinal morphometry. It can be concluded that white-feathered quails had, in general, higher values of productivity compared with the brown-feathered ones during growing and laying periods.

## Introduction

Japanese quails (*Coturnix japonica*) are promising poultry species that belong to the order *Galliformes* and the family *Phasianidae*, like the chicken (Minvielle, 2004; Batool et al., 2021). Despite their small size, quail are reared for meat and egg production, especially in low-resource rural areas as a valuable and cheap source of animal protein (Vali, 2008; Inci et al., 2015). Moreover, quails are used as an animal model for biological and genetic studies in poultry because of their small body size, small required floor space for a large number of birds, small generation interval, resistance to infection, and high growth rate (Minvielle et al., 2007; Tarhyela et al., 2012). The different plumage colors in quails were found to be associated with different mutations in different genes, such as MC1R (Kageyama et al., 2018), AISP (Li et al., 2019), and PMEL genes (Ishishita et al., 2018). Due to the recessive plumage-color mutations, different varieties of Japanese quails have been recognized such as mottled brown-feathered and white-feathered quails (Truax and Johneson, 1979; Minvielle et al., 2002) with significant impact on growth performance parameters (Minvielle et al., 1999, 2005, 2007; Petek et al., 2004). In this regard, the recessive white color mutation is associated with less body weight compared to the wild-type Japanese quail (Petek et al., 2004; Minvielle et al., 2005). However, compared to the brown type of Japanese quail, the white type showed higher body weight and lower feed conversion ratio, especially in the 4^th^ and 5^th^ weeks of age (Inci et al., 2015). Therefore, the white-feathered Japanese quails recently have been favored for meat production purposes (Islam et al., 2014). Additionally, because of the better egg weight, chick weight, and hatchability rate in the case of white-feathered Japanese quails compared to their counterparts (Islam et al., 2014), along with the lower chick price, at the commercial level, there is an increasing spread of white-feathered Japanese quails compared to other types of Japanese quails has been noticed.

The quality and composition of poultry meat are influenced by numerous factors including genotype and age (Genchev et al., 2005). In this context, an increment in protein, fat, and caloric value by increasing the age of farmed birds is usually found (Khalifa et al., 2016). Thus, despite poultry meat and eggs are consider good sources of good quality animal protein, their fat contents affect human health. High cholesterol and total fat contents of poultry meat influence human health as considered cardiovascular risk factors (Milićević et al., 2014). Therefore, due to the lower fat and cholesterol contents of quail meat compared to other poultry meat, it is more recommended for humans (Khalifa et al., 2016).

Previous studies listed only the differences in growth performance and carcass traits between white and brown-feathered Japanese quail. In addition, there are no reports neither for the fat content in carcasses and eggs nor the physiological, hematological, and intestinal histological features of these two varieties. Therefore, this study aimed to investigate the characteristic differences between the white and brown-feathered Japanese quails, by evaluating the carcass traits and egg fat content at 4^th^, 5^th^, and 6^th^ weeks of age, blood parameters and intestinal histopathological features.

## Material and methods

### Birds management and diets

A total of 1200 one-day-old Japanese quail (*Coturnix japonica)* chicks of two varieties (brown and white-feathered) were used in this study. Chicks of the same variety were numbered, randomly allocated into 4 replicates (150 chicks/replicate), and housed in a deep litter system with the housing temperature adjusted at 35 to 37°C for the first week, and then gradually decreased by age to reach 24 to 26°C, with relative humidity ranging from 50 to 70% throughout the experiment. Chicks were supplied with ad libitum fresh water and a commercial diet of 23% crude protein (CP) and 3100 kcal/kg metabolizable energy (ME).

Individual live body weights were determined every week. Feed intake was also calculated every week by dividing total feed consumption by the number of birds in each cage, and to avoid the biased calculation for feed intake, if a bird died in a cage during any week, the feed intake was calculated immediately including the dead and live birds. At the 4^th^ week of age, 80 birds from each variety (20 birds/replicate, 10 males and 10 females) were randomly selected and slaughtered.

At six weeks of age, birds were transferred into a cage system (30× 30×25cm, L×W×H) in which birds were housed at a sex ratio of 2 females: 1 male. Birds were fed a commercial layer-quail diet of 21% crude protein (CP) and 2800 kcal/kg (ME), and provided with ad libitum fresh water through a nipple water delivery system. Eggs were collected daily, and egg production was expressed on a bird-day basis. Individual egg weights were recorded to calculate the mean egg weight for all experimental periods. Then, the total egg mass was calculated by multiplying egg weights by egg production.

### Meat quality and carcass characteristics assessment

Before slaughtering, live body weights (BW) were recorded. After slaughter, body weight after bleeding, body weight after de-feathering, and hot carcass weight were recorded. Moreover, some visceral organs were weighed like the spleen, gizzard, liver, heart, pancreas, intestine, reproductive organs (ovary, testis, and oviduct), and total body fat weight (skin plus abdominal fat). Variation in abdominal fat weight has been noticed at the 4^th^ week of age among the two varieties of Japanese quail and between males and females. So, subsequent assessments of the abdominal fat contents were done at the 5^th^ and 6^th^ weeks of age to determine whether the fat content changes with increasing age.

Carcass traits also include weight (g), length (g), and width (cm) of breast muscle, thigh muscle weight (g), and length of the tibia bone (cm) were measured. Additionally, meat quality characteristics such as meat color, water-holding capacity, tenderness, and pH were evaluated, at 24 h post-mortem, in an internal section of the left pectoral major muscle. Data were obtained within 45 min post-mortem using a chromameter (Minolta ChromaMeter CR-300, Minolta Inc., Osaka, Japan) and pH direct measuring apparatus (pH-STAR, R.Matthaus, Berlin, Germany) with three repeats for each sample. The color intensity of meat extract was determined according to the method mentioned by Yamazaki (1981). Ten grams of meat sample were taken with 22.5 ml distilled water in a dark room for 10 min; the color intensity (absorbance) of the filtrate was measured at 542 nm by Spectro. Nic 21VUFA.

### Biochemical analysis

At the 6^th^ week of age, blood samples (20 birds/replicate, 10 males and 10 females) were withdrawn into tubes without anticoagulant, centrifuged at 3000 rpm for 10 min to separate serum, and stored at −20oC for further analysis. Serum triiodothyronine (T3), thyroid-stimulating hormone (TSH), growth hormone (GH), total proteins, albumin, globulin, lipid profile (total lipids, total cholesterol, HDL- cholesterol and LDL-cholesterol), urea, creatinine, calcium, and phosphorous. Also, the activity of serum alanine aminotransferase (ALT), aspartate aminotransferase (AST), and alkaline phosphatase (ALP) was calorimetrically determined using an automatic analyzer (Chemwell®, Megazyme, Wicklow, Ireland), and commercial kits (Labtest Diagnóstica S.A, Lagoa Santa, Brazil). Globulin was calculated as the difference between serum total proteins and albumin.

### Histopathological examination of liver and intestines

Random representative Jejunum tissue specimens were fixed in 10% formalin solution for 24 h. Specimens were embedded in paraffin at 56°C for 24 h. Paraffin tissue blocks were prepared for sectioning at 5 microns by rotatory microtome. The tissue sections were collected on glass slides, deparaffinized and stained by hematoxylene and eosin (H&E) according to the method described previously (Bancroft et al., 1994). Then they were examined for villi length, crypt depth, and villi width in mm using a light microscope. Three fields per slide and 5 villi and crypts per field were measured. Villi length, crypt depth, and villi width were measured using Image J software, the measurements were done as described previously (Caruso et al., 2012). Briefly, the villi length was measured from its basal region, which coincides with the top of the crypts. The depth of the crypt (DC) was measured from its base to the crypt-villus transition. These were measured on both sides (right and left) of the cut, to reduce, by averaging, any changes in measurements and were statistically analyzed.

### Egg quality assessment

One hundred eggs were randomly collected for three consecutive weeks from each quail variety to determine egg yield characteristics. External egg quality traits (egg weight, shape index, shell thickness, shell percentage) and internal traits (albumen percentage, albumen index, yolk percentage, yolk index, and Haugh unit) were measured. Egg length and width were measured using a 0.01 mm digital display caliper and the shape index was calculated. To determine the internal characteristics of the eggs, all the eggs were cracked then their contents were removed. Albumen length, width, and height in addition to yolk width, and height were measured using the digital caliper. The shell thickness and weight were also determined. The shell with the membranes and the yolk was separated from the albumen and weighed, and the relative proportions were determined. Albumen weight was calculated by subtracting the yolk and shell weight from the total egg weight. Shell’s thickness was measured in three equatorial regions of each shell using a manual micrometer. Yolk color was determined using the Roche Color Fan (DSM Yolk Color Fan, Basel, Switzerland). Shell, albumen, and yolk percentages were calculated as a ratio of egg weight. Haugh units were calculated based on egg weight and albumen height.

### Statistical analysis

Data were analyzed using the GLM procedure of SAS 9.2 (SAS Institute Inc., 2008). All the traits during the fattening and laying periods were analyzed using the following model: *Y*_*ijk*_*= μ+ L*_*i*_ *+ S*_*j*_ *+ (LS)*_*ij*_ *+ e*_*ijk*_ where μ is the general mean, *Y*_*ijk*_ represents the dependent variable, *L*_*i*_ is the effect of the quail variety, *S*_*j*_ is the effect of sex, *(LS)*_*ij*_ is the interaction between line *i* and sex *j*, and *e*_*ijk*_ is the residual effect. The different levels of each effect included in the models were compared by using Duncan's multiple range test where significance levels were detected as first-class error at *P* = 0.05. For reproductive organs and egg quality traits, the previous model was applied but with excluding the sex effect, *Y*_*ij*_*= μ+ L*_*i*_ *+ e*_*ij*_ where μ is the general mean, *Y*_*ij*_ represents the dependent variable, *L*_*i*_ is the effect of the quail variety and *e*_*ij*_ is the residual effect.

## Results

### Growth performance and carcass characteristics in the two Japanese quail varieties

Body weights, growth rate, feed intake, and feed conversion ratio (FCR) from hatch to six weeks of age in the two quail varieties are presented in Table 1. Compared with the brown-feathered quails, the white-feathered quails had significantly heavier body weights with higher growth rates from hatch to the 6^th^ week of age (*p* < 0.05). The higher growth performance of white-feathered quails was associated with increased feed intake compared with brown-feathered ones (*p* < 0.05). However, there were no significant differences between the two quail varieties at the FCR level (*p* > 0.05).

**Table 1:** Growth performance of the brown- and white-feathered quails

Variables	Brown-feathered	White-feathered	*P* value
BW0	10.10±0.09^b^	12.47±0.08^a^	0.0010
BW1	24.06±0.15^b^	28.50±0.21^a^	0.0001
BW2	45.30±0.15^b^	59.05±0.29^a^	0.0020
BW3	98.12±0.73^b^	117.67±0.62^a^	0.0001
BW4	156.94±0.94^b^	193.30±1.08^a^	0.0001
BW5	195.45±1.1^b^	246.38±1.30^a^	0.0001
BW6	236.40±1.30^b^	291.95±1.25^a^	0.0001
GR_0-2	2.65±0.02^b^	3.32±0.01^a^	0.0001
GR2_2-4	7.90±0.07^b^	9.94±0.09^a^	0.0001
GR_0-4	5.13±0.30^b^	6.63±0.03^a^	0.0001
GR_0-6	3.98± 0.05^b^	4.9± 0.03^a^	0.0001
FI_0-2	81.97±0.46^b^	105.59±0.37^a^	0.0001
FI_2-4	241.11±0.60^b^	320.98±0.50^a^	0.0001
FI_0-4	399.48±0.85^b^	522.43±0.91^a^	0.0001
FI_0-6	615.19±1.19^b^	791.23±1.24^a^	0.0001
FCR_0-2	2.28±0.20	2.32±0.15	0.980
FCR_2-4	2.26±0.20	2.37±0.12	0.580
FCR_0-4	2.76±0.21	2.85±0.17	0.660
FCR_0-6	2.80±0.27	2.89±0.25	0.510

Traits of different quial varities with different superscripts are significantly different (*P* < 0.05)

The carcass characteristics of the two studied varieties at the fourth week of age are presented in Table 2. It can be observed that live body weight, weight after slaughtering, and weight after defeathering were affected by the interaction between strain and sex. As the females of white feather quails were significantly superior (*p* < 0.05) to other groups in the three above-mentioned traits. For carcass weight trait, the interaction effect was insignificant, and the trait was significantly affected by both stain and sex, where females and white feathered quails had significantly higher (*p* < 0.05) carcass weight than males and brown-feathered quails. Gizzard weight was also influenced by the interaction between strain and sex. While the difference in in the weights of heart, spleen, pancreas, intestine, and liver were not significant. Interesting differences in total body fat were noticed among the two quail varieties. The total body fat weight was significantly higher in white-feathered quails compared to brown-feathered ones (*p* < 0.05), without sex differences in the same quail variety. Since previous studies reported (Abou-Kassem et al., 2019) changes in lipid levels with age, the later finding was expected to change with increasing bird age. So, subsequent further assessments of carcass yields, and total body fat were performed at the 5^th^ and 6^th^ weeks of age (Tables 3 and 4)

**Table 2:** Carcass characteristics at the 4^th^ week of age

St	Gn	live weight (g)	after slaughter	after defeathering	Carcass weight (g)	spleen (g)	Gizzard (g)	Heart (g)	Pancreas (g)	Liver (g)	Intestine (g)	Total body fat (g)
B	F	192.8±5.45^b^	188.6±6.26^b^	176.6±6.36^b^	132.8±4.89^b^	0.29±0.07	4.24±0.41^b^	1.5±0.06	0.75±0.09	6.05±0.28	12.69±0.84^b^	10.91±0.64^b^
B	M	192.6±4.06^b^	186±4.19 ^b^	178.8±4.46^b^	126.4±4.52^b^	0.21±0.02	4.82±0.31^ab^	1.46±0.06	0.66±0.06	6.23±0.28	12.6±0.44^b^	10.56±1.02^b^
W	F	229.8±7.04^a^	221.8±7.32^a^	207.8±7.27^a^	153.6±6.41^a^	0.25±0.03	5.74±0.20^a^	1.62±0.07	0.80±0.06	7.14±0.47	16.29±1.27^a^	13.14±0.93^a^
W	M	195.2±6.44^b^	187.8±6.76^b^	176.6±6.57^b^	132.4±4.26^b^	0.22±0.02	3.98±0.72^b^	1.53±0.12	0.68±0.04	6.41±0.61	12.85±1.44^b^	11.61±0.72^b^
Quail strain												
B		192.7±3.2^b^	187.3±3.58^a^	177.7±3.68^a^	129.6±3.31^b^	0.25±0.04	4.53±0.26	1.48±0.04	0.70±0.05	6.14±0.19	12.65±0.44	10.73±0.57^b^
W		212.5±7.31^a^	204.8±7.36^b^	192.2±6.95^b^	143±5.06^a^	0.23±0.02	4.86±0.46	1.58±0.06	0.74±0.04	6.77±0.38	14.57±1.07	12.37±0.61^a^
Gender												
F		211.3±7.46^a^	205.2±7.15^a^	192.2±6.91^a^	143.2±5.14^a^	0.27±0.04	4.99±0.33	1.56±0.05	0.78±0.05	6.59±0.31	14.49±0.93	12.02±0.65
M		193.9±3.61^b^	186.9±3.76^b^	177.7±3.76^b^	129.4±3.09^b^	0.21±0.01	4.4±0.39	1.5±0.065	0.67±0.03	6.32±0.31	12.73±0.71	11.08±0.61
Source of variation, P value												
St		0.004	0.011	0.031	0.023	0.665	0.470	0.247	0.610	0.161	0.007	0.007
Gn		0.009	0.009	0.033	0.013	0.253	0.213	0.454	0.144	0.532	0.120	0.280
St*Gn		0.009	0.022	0.011	0.164	0.620	0.022	0.784	0.872	0.310	0.130	0.490

*B* brown-feather quails; *W* white-feather quails; *F* females; *M* males; *St* strain; *Gn* gender

Traits of different quial varities with different superscripts are significantly different (*P* < 0.05)

**Table 3:** Carcass characteristics at the 5^th^ week of age

St	Gn	live weight (g)	after slaughter	after defeathering	Carcass weight (g)	spleen (g)	gizzard(g)	Heart (g)	Pancreas (g)	Liver (g)	Intestine (g)	ovary	oviduct	**testis**	**Total body fat (g)**
B	F	256.2±6.29^ab^	249±5.49^ab^	239.4±5.97^ab^	183.89±5.25	0.366±0.07	4.88±0.37	2.48±0.14	0.857±0.06	8.2672±0.75^b^	13.9±0.699	1.45±0.46^b^	1.51±0.48	----------	16.89±1.34
	M	242.0±4.41^b^	235.6±4.34^b^	227.2±4.30^a^	174.10±4.35	0.175±0.01	4.66±0.15	2.05±0.07	1.001±0.23	6.758±0.56^b^	13.34±0.81	-------------	---------	3.34±0.39^b^	19.1±1.085
W	F	271.8±5.47^a^	266.2±4.96^a^	252.8±5.27^a^	183.69±7.28	0.419±0.19	5.49±0.23	3.01±1.10	0.855±0.06	12.2576±2.39^a^	13.94±0.65	6.67±1.65^a^	6.68±2.31	---------	17.89±2.25
	M	259.8±7.75^ab^	252.8±7.83^ab^	241.8±6.98^ab^	185.29±6.77	0.372±0.04	4.98±0.27	2.43±0.16	0.837±0.04	6.521±0.41^b^	13.16±0.90	-------------	--------	6.38±1.13^a^	19.89±1.41
Quail Strains															
B		249.1±4.32^b^	242.3±3.98^b^	233.3±4.04^b^	178.99±3.6	0.27±0.04	4.77±0.19	2.27±0.10	0.929±0.11	7.5126±0.50	13.62±0.51	-------------	-------	---------	17.99±0.89
W		265.8±4.9^a^	259.5±4.9^a^	247.3±4.51^a^	184.49±4.69	0.396±0.09	5.23±0.19	2.72±0.53	0.846±0.03	9.3893±1.49	13.55±0.54	------------	--------	----------	18.89±1.29
Gender															
F		264±4.71^a^	257.6±4.51^a^	246.1±4.37	183.79±4.23	0.393±0.09	5.19±0.23	2.75±0.53	0.856±0.04	10.2624±1.35^a^	13.92±0.45	--------------	---------	-------------	17.39±1.24
M		250.9±5.14^b^	244.2±5.1^b^	234.5±4.58	179.69±4.22	0.273±0.04	4.82±0.15	2.24±0.10	0.919±0.11	6.6395±0.33^b^	13.25±0.57	-------------	---------	------------	19.49±0.85
Source of variation, P value															
St		0.011	0.009	0.021	0.371	0.257	0.101	0.430	0.531	0.161	0.923	0.021	0.061	0.033	0.571
Gn		0.041	0.031	0.058	0.233	0.281	0.187	0.381	0.633	0.011	0.401	---------	---------	---------	0.200
St*Gn		0.853	1.000	0.911	0.262	0.510	0.581	0.892	0.533	0.124	0.890	---------	---------	---------	0.941

*B* brown-feather quails; *W* white-feather quails; *F* females; *M* males; *St* strain; *Gn* gender

Traits of different quial varities with different superscripts are significantly different (*P* < 0.05)

**Table 4:** Carcass characteristics at 6^th^ week of age

St	Gn	live weight (g)	after slaughter	after defeathering	Carcass weight (g)	Spleen (g)	Gizzard (g)	Heart (g)	Pancreas (g)	Liver (g)	Intestine (g)	Ovary (g)	Testis (g)	**Oviduct (g)**	**Total body fat (g)**
B	F	269±5.61^ab^	260.8±5.01^b^	250.8±7.24^b^	179.2±3.24^ab^	0.27±0.08	3.936±0.25	2.10±0.10	0.75±0.06^ab^	8.27±0.96^a^	14.35±1.38^a^	9.09±2.71	---------	9.99±1.13	16.6±1.36^b^
	M	242.2±4.37^c^	235.4±5.40^c^	226.6±4.26^b^	173.2±4.68^b^	0.27±0.04	4.199±0.36	2.18±0.06	0.79±0.06^ab^	5.48±0.24^b^	11.31±0.4^b^	---------	5.58±0.87^b^	-----------	15.4±1.74^b^
W	F	292.8±6.93^a^	287.4±7.73^a^	276.4±8.62^a^	190.8±7.00^ab^	0.66±0.26	4.543±0.33	2.10±0.12	0.85±0.07^a^	8.8±0.39^a^	14.15±0.52^a^	13.31±0.63	---------	11.94±1.05	21.4±0.92^a^
	M	266.6±12.51^b^	262.6±12.13^b^	250.6±1.12^b^	196.2±10.7^a^	0.30±0.07	3.692±0.34	2.42±0.13	0.60±0.06^b^	5.14±0.45^b^	10.25±0.41^b^	-----------	9.06±0.75^a^	-----------	25.2±2.1^a^
Quail Strains															
B		255.6±5.58^b^	248.1±5.47^b^	238.7±5.65^b^	176.2±2.86^b^	0.27±0.04	4.06±0.21	2.14±0.05	0.77±0.04	6.87±0.66	12.83±0.84	-----------	-------	-----------	16.0±1.06^b^
W		279.7±8.03^a^	275±7.94^a^	263.5±7.90^a^	193.5±6.09^a^	0.48±0.14	4.11±0.26	2.26±0.10	0.73±0.06	6.97±0.67	12.2±0.72	---------	-----------	-----------	23.3±1.25^a^
Gender															
F		280.9±5.78^a^	274.1±6.20^a^	263.6±6.81^a^	185±4.12	0.46±0.14	4.23±0.22	2.10±0.07	0.80±0.04	8.53±0.49^a^	14.25±0.69^a^	----------	-----------	-----------	19.0±1.11
M		254.4±7.45^b^	249±7.73^b^	238.6±6.89^b^	184.7±6.70	0.29±0.04	3.94±0.24	2.30±0.08	0.70±0.05	5.31±0.25^b^	10.78±0.32^b^	----------	----------	-----------	20.3±2.08
Source of variation, P value															
St		0.008	0.004	0.008	0.022	0.160	0.869	0.284	0.51	0.871	0.441	0.164	0.011	0.242	0.001
Gn		0.004	0.006	0.007	0.958	0.237	0.371	0.093	0.135	0.0001	0.001	---------	---------	---------	0.421
St*Gn		0.970	0.951	0.920	0.421	0.241	0.101	0.301	0.041	0.462	0.588	---------	---------	---------	0.130

*B* brown-feather quails; *W* white-feather quails; *F* females; *M* males; *St* strain; *Gn* gender

Traits of different quial varities with different superscripts are significantly different (*P* < 0.05)

At five weeks of age (Table 3), live body weights, weight after slaughtering, and weight after defeathering traits were affected by both sex and strain, in the absence of significant interaction between them. The females and white-feathered quails had significantly higher live body weights, weights after slaughter, and after de-feathering compared with males and brown-feathered quails (*p* < 0.05). While, carcass weight, heart, gizzard, spleen intestine, pancreas, and total body fat did not reveal any significant changes, Liver weight, however, was affected by sex only, as females had higher liver weights than males. Also, there was a significant effect for strain on both ovary and testis weights. Likewise, at the sixth week of age (Table 4), there were significant effects for strain and sex on live body weights, weight after slaughtering, and weight after defeathering. Females and white-feather quails showed significant increases in live body weight, and weight after slaughter and defeathering (*p* < 0.05). For the carcass weight, the effect of strain was significant, but both stain by sex and sex effects were insignificant. It can be noticed that the white-feather quails had higher body weights than the brown-feather quails (*p* < 0.05). Collectively, the white feather quails showed higher live body weight, weights after slaughter, and de-feathering and carcass weight compared with the brown feather quails (*p* < 0.05). Moreover, the females showed significant increases in liver, and intestines weights compared with males (*p* < 0.05). The pancreas weight was affected by the interaction between sex and strain. For testis weights, the males of white-feather quails exhibited higher weights compared with those of the brown-feather variety (*p* < 0.05). Also, there was a significant effect for strain on total body fat weight (*p* < 0.05), as white-feathered quails had higher total body fat weight than brown-feathered ones.

### Meat quality of the two quail varieties

Meat quality characteristics including water-holding capacity, color, tenderness, pH, breast muscle weight, breast muscle length, breast muscle width, thigh weight, and tibia length were reported in Table 5. For water holding capacity and meat tenderness, statistically significant effects of quail variety were found (*p* < 0.05), without significant effects of sex and variety-sex interaction. The brown variety recorded a higher water-holding capacity and tenderness compared with the white-feathered one (*p* < 0.05). Regarding the meat color, there was a statistically significant effect of quail sex (*p* < 0.05) without statistical significance for either quail variety or quail variety-sex interaction (*p* > 0.05). Males exhibited the highest color index in comparison with females (*p* < 0.05). Moreover, meat pH values displayed statistical significance for quail variety, sex, and their interaction (*p* < 0.05). Males and females of white quails along with females of brown ones showed the highest pH values compared to males of brown quails (*p* < 0.05). Generally, the white variety had higher pH values compared to the brown one, in addition, the females of both varieties showed higher values compared to males (*p* < 0.05).

**Table 5:** Meat quality characteristics at 6 weeks

St	Gn	WHC	Color	Tenderness	pH	BM weight (g)	BM length (g)	BM width (cm)	thigh weight (g)	tibia length (cm)
B	F	9.86±0.21^a^	3.12±0.38^ab^	6.74±0.38^a^	7.22±0.11^a^	53.6±2.03^a^	6.79±0.11^a^	4.73±0.24	66.6±1.5^ab^	9.97±0.10^ab^
	M	9.48±0.51^a^	3.70±0.29^a^	5.78±0.78^a^	6.03±0.02^b^	45.60±1.2^b^	6.35±0.11^b^	4.60±0.12	62.2±1.82^b^	9.30±0.18^b^
W	F	7.30±0.55^b^	2.50±0.39^b^	4.8±0.61a^b^	7.07±0.07^a^	51.2±3.16^ab^	6.78±0.14^a^	4.26±0.15	66.6±2.03^ab^	10.06±0.27^a^
	M	7.46±0.60^b^	3.84±0.24^a^	3.62±0.84^b^	7.322±0.16^a^	49.2±1.74^ab^	6.8±0.14^a^	4.43±0.13	69.6±2.78^a^	9.92±0.18^ab^
Quail Strains										
B		9.67±0.27^a^	3.41±0.24	6.26±0.44^a^	6.62±0.20^b^	49.60±1.73	6.57±0.10	4.67±0.13	64.4±1.33	9.67±0.18
W		7.38±0.38^b^	3.17±0.31	4.21±0.52^b^	7.19±0.09^a^	50.20±1.73	6.79±0.09	4.34±0.09	68.1±1.70	9.99±0.15
Gender										
F		8.58±0.51	2.81±0.27^b^	5.77±0.47	7.15±0.06^a^	52.4±1.82^a^	6.78±0.09	4.49±4.49	66.6±1.19	10.02±0.13
M		8.47±0.50	3.77±0.18^a^	4.7±0.65	6.67±0.23^b^	47.4±1.16^b^	6.57±0.11	4.52±4.52	65.9±1.99	9.65±0.15
Source of variation, P value										
St		0.0003	0.480	0.008	0.0001	0.777	0.111	0.081	0.088	0.121
Gn		0.820	0.011	0.130	0.0005	0.031	0.128	0.888	0.742	0.071
St*Gn		0.593	0.272	0.872	0.0001	0.180	0.101	0.391	0.092	0.262

*B* brown-feather quails; *W* white-feather quails; *F* females; *M* males; *St* strain; *Gn* gender; *WH* water holding capacity; *BM* breast muscle

Traits of different quial varities with different superscripts are significantly different (*P* < 0.05)

For breast muscle weight, only sex had a significant effect (*p* < 0.05) without statistical significance for variety, where females showed higher weight compared with the males (*p* < 0.05). Breast muscle length and width did not show any significant changes. Likewise, the thigh muscle weight and tibia length did not show any significant changes.

### Serum biochemical characteristics

The effects of Japanese quail variety, sex, and their interaction on serum biochemical characteristics are presented in Table 6. Non-significant changes in the serum concentration of ALT, total protein, and ALP in the two-studied quail varieties were noticed. While, for AST, cholesterol, and triglycerides, there was a significant effect of sex (*p* < 0.05). Males displayed the highest values of AST and cholesterol but in the case of triglycerides, the females showed the highest values in comparison with males. Albumin concentration was significantly affected by quail variety, sex, and their interaction (*p* < 0.05). Generally, the brown-feathered males had low albumin levels compared with other groups (*p* < 0.05). For globulin, the interaction between sex and variety showed a significant effect, where the brown-feather females displayed less globulin concentration compared with the others (*p* < 0.05). The level of high-density lipoprotein (HDL) was significantly affected by the interaction between sex and variety, without significant effects for sex or variety independently. The highest value was reported for males of brown-feather quails compared with the others. Low-density lipoprotein (LDL) was significantly (*p* < 0.05) influenced by both sex, and sex by variety interaction The significantly higher concentrations of LDL were noticed in the case of males and females of the white feather quail compared with males and females of the brown-feather quail (*p* < 0.05). For kidney function indicators, creatinine, and urea, they did not exhibit any significant changes between the two varieties. Calcium and phosphorus ion concentrations were affected by the sex of the birds (*p* < 0.05), where females had significantly higher concentrations compared with males.

**Table 6:** Blood biochemical markers at 6 weeks

St	Gn	ALT (U/L)	AST (U/L)	Albumin (g/dl)	Total protein (g/dl)	Globulin	ALP(U/L)	Cholesterol (mg/dl)	Triglyceride
B	F	18.5±1.16	234.6±32.0^ab^	1.32±0.20^a^	2.66±0.26	1.33±0.18^b^	1179.7±100.52	103.8±17.51^b^	231.3±61.36^a^
	M	28.8±0.91	264.0±24.8^ab^	0.51±0.07^b^	2.75±0.38	2.23±0.36^ab^	1176.8±99.63	84.1±9.49^b^	49.5±5.85^b^
W	F	19.4±1.67	197.3±17.3^b^	1.37±0.09^a^	4.17±0.62	2.79±0.75^a^	1040.6±113.62	67.7±10.88^b^	211.3±61.41^a^
	M	16.7±0.84	316.2±34.8^a^	1.40±0.14^a^	3.02±0.59	1.61±0.50^ab^	1014.7±121.67	142.9±11.98 ^a^	101.8±18.41^ab^
Quail Strains									
B		23.6±5.47	249.3±20.	0.91±0.14^b^	2.70±0.22	1.78±0.22	1178.25±68.88	93.95±9.95	140.4±36.53
W		18.0±0.96	256.7±23.3	1.38±0.08^a^	3.59±0.44	2.20±0.39	1027.65±81.07	105.3±11.68	156.55±33.63
Gender									
F		18.9±0.99	215.9±81.5^b^	1.34±0.11^a^	3.41±0.37	2.06±0.33	1110.15±75.53	85.75±10.85^b^	221.3±42.31^a^
M		22.7±5.50	290.1±97.0^a^	0.95±0.12^b^	2.88±0.34	1.92±0.31	1095.75±78.76	113.5±10.04^a^	75.65±11.15^b^
Source of variation, P value									
St		0.32	0.79	0.002	0.07	0.34	0.17	0.38	0.71
Gn		0.49	0.01	0.008	0.29	0.74	0.89	0.03	0.002
St*Gn		0.25	0.12	0.005	0.21	0.02	0.91	0.0007	0.42
St	Ge	HDL	LDL	creatinine	urea	Ca T	Ca ion	phosphorus	
B	F	54.40±1.72^ab^	3.14±15.8^c^	0.28±0.02	15.1±1.01	17.19±1.43^a^	1.90±0.11^a^	12.84±2.8^a^	
	M	57.91±1.01^a^	16.30±8.7^b^	0.23±0.02	13.5±1.07	11.24±0.87^bc^	1.37±0.10^b^	8.91±0.77^ab^	
W	F	55.0±2.23^ab^	29.56±16.7^a^	0.27±0.01	15.0±1.05	15.88±2.51^ab^	1.81±0.24^ab^	11.41±0.8^ab^	
	M	51.61±1.63^b^	70.94±15.5^a^	0.27±0.01	15.0±0.88	10.80±1.45^c^	1.32±0.18^b^	7.44±0.9^b^	
Quail Strains									
B		56.15±1.05	9.72±8.94	0.26±0.01	14.3±0.74	14.21±1.06	1.63±0.09	10.87±1.48	
W		53.30±1.40	20.69±16.01	0.27±0.01	15.0±0.66	13.34±1.52	1.57±0.15	9.42±0.74	
Gender									
F		54.70±1.37	13.21±11.82^b^	0.28±0.01	15.0±0.71	16.53±1.41^a^	1.85±0.13^a^	12.12±1.43^a^	
M		54.75±1.18	43.62±10.7^a^	0.25±0.01	14.2±0.69	11.02±0.82^b^	1.35±0.10^b^	8.17±0.6^b^	
Source of variation, P value									
St		0.10	0.45	0.55	0.49	0.60	0.69	0.36	
Gn		0.97	0.004	0.22	0.43	0.002	0.004	0.01	
St*Gn		0.04	0.004	0.22	0.43	0.79	0.91	0.98	

*B* brown-feather quails; *W* white-feather quails; *F* females; *M* males; *St* strain; *G* gender

Traits of different quial varities with different superscripts are significantly different (*P* < 0.05)

### Egg production characteristics

The hen-day egg production (HDEP) and egg quality characteristics of the two studied quail varieties, white and brown-feathered were evaluated (Table 7). No significant differences were noticed between the two quail varieties in terms of HDEP, egg weight, yolk percentage, yolk index, yolk color, albumen index, Haugh unit, and albumen height. However, there were significant variations between brown and white-feathered quails in terms of egg shape index, shell thickness and percentage of relative shell weight. The higher eggshell weight and thickness were reported for white-feathered quails compared to the brown-feathered ones (*p* < 0.05).

**Table 7:** Egg characteristics of brown-feather and white feather quails

	HDEP	Egg weight	Shape index	Shell thickness	Shell %	Yolk %	Yolk index	Yolk color	Albumin %	Albumin index	Haugh unit	Albumin height
B	75.5±4.20	14.93±0.12	77.1±0.50^b^	205±0.4.3^b^	10.45±0.29^b^	32.08±0.49	46.23±1.6	4.93±0.12	57.46±0.59^a^	6.75±0.23	79.62±1.24	5.90±0.17
W	70.12 ±3.50	15.34±0.27	78.81±0.49^a^	218.66±4.9^a^	11.74±0.25^a^	33.64±0.75	46.13±1.03	5.03±0.12	54.61±0.79^b^	6.88±0.27	80.26±1.47	5.81±0.19
P value	0.23	0.17	0.04	0.04	0.004	0.08	0.95	0.57	0.005	0.74	0.74	0.72

*B* brown-feather quails; *W* white-feather quails

Traits of different quial varities with different superscripts are significantly different (*P* < 0.05)

### Intestinal morphological characteristics of white and brown-feathered quail

**Fig. 1:**
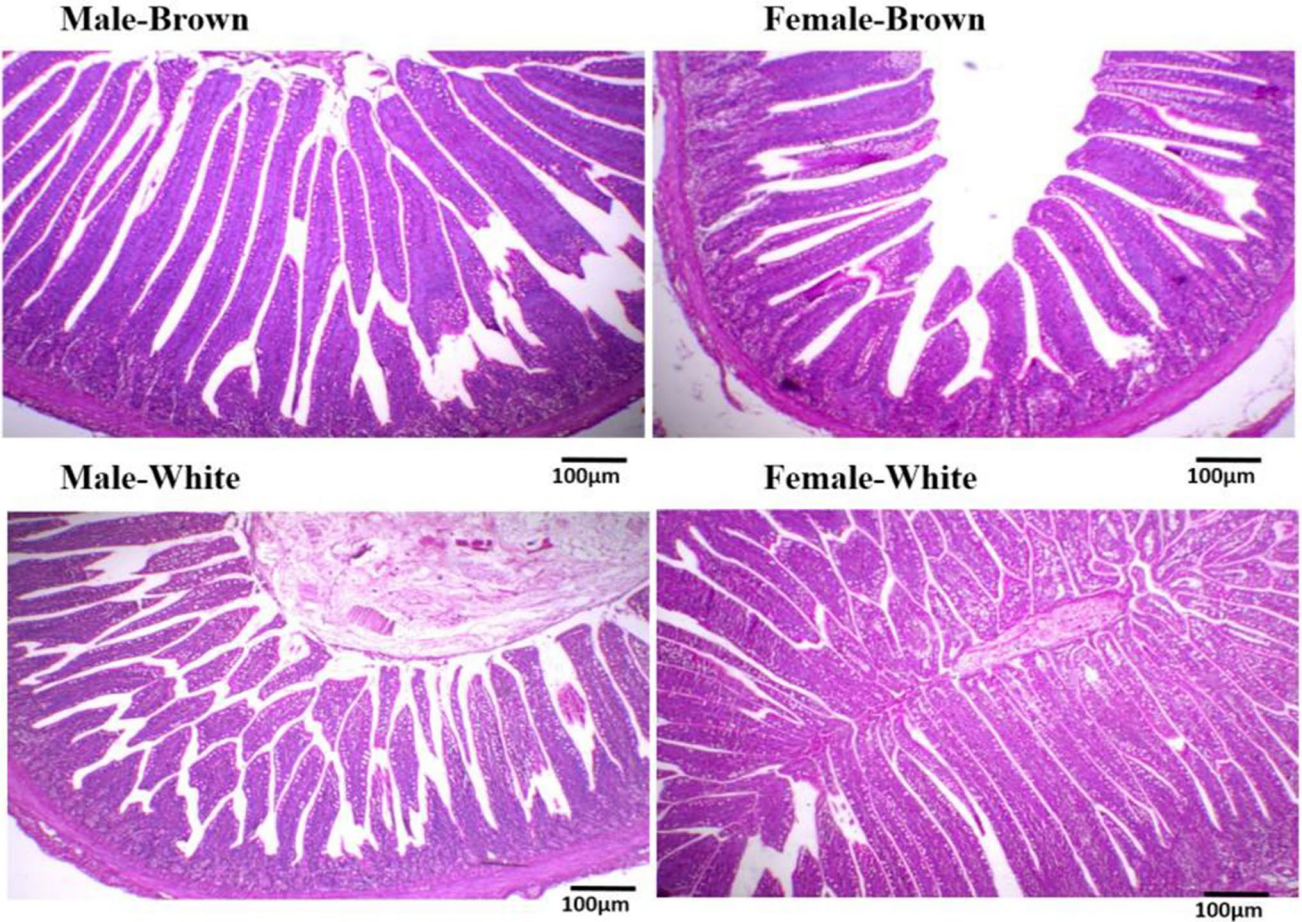
Morphology of middle part of intestine (jejunum) of brown- and white-feathered Japanese quail varieties at the end of the growing period

**Fig. 2:**
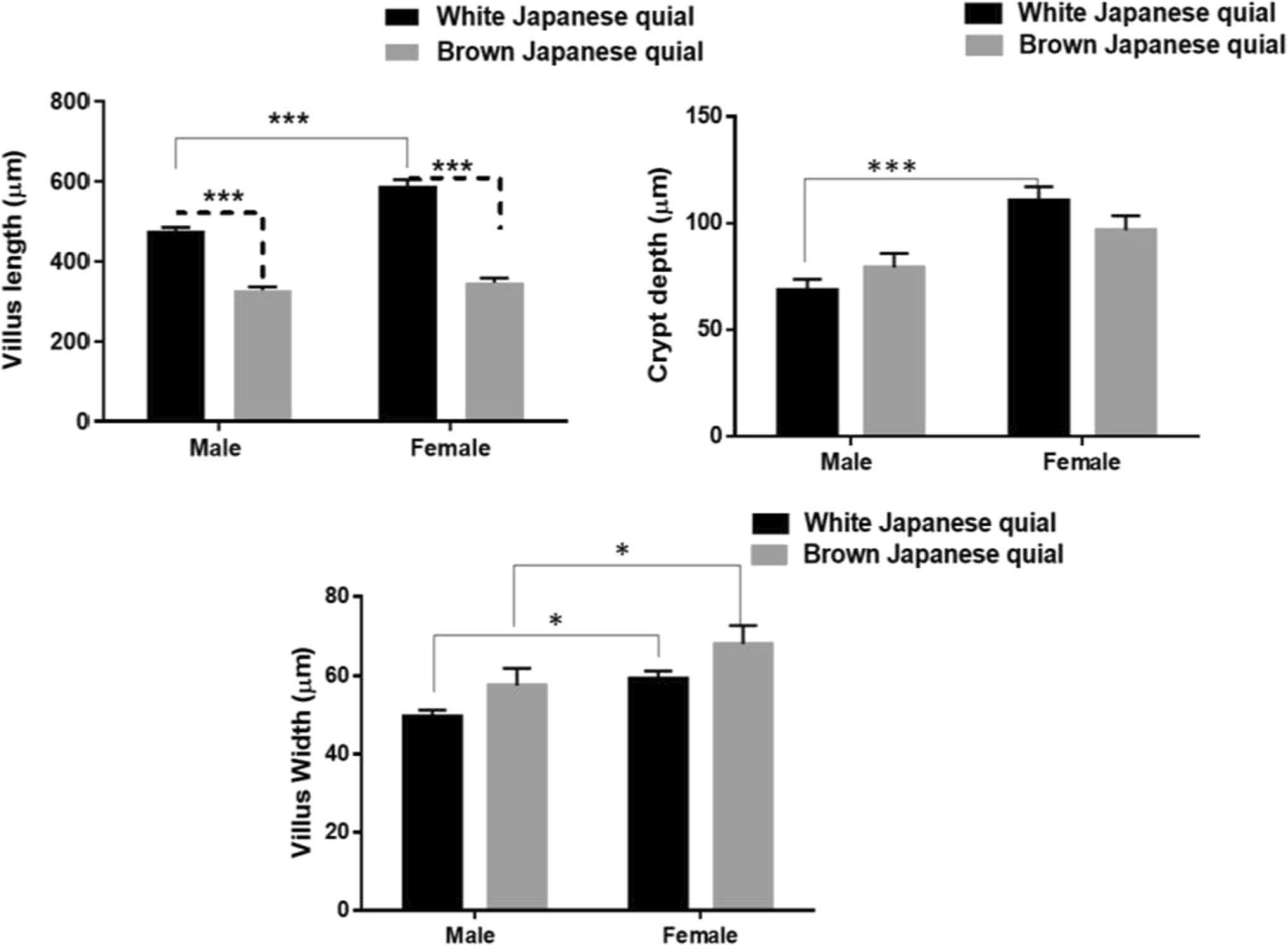
Villi length, villi depth, and crypt depth in males and females of brown- and white-feathered quails

Figures 1 and 2 represent the morphology of the middle part of the intestine (jejunum) of brown and white-feathered Japanese quail varieties at the end of the growing period (at 6 weeks old). Figure 1 illustrates the jejunum architecture of males and females of the two quail lines. Normal intestinal morphology including intact villi lined with goblet cells, normal intestinal glands in the lamina propria, tunica muscularis, and tunica serosa were found in males and females of the two quail varieties. Significant higher villi lengths were measured in both males and females of the white-feather quail variety compared to the brown-feather one (Fig. 2). Also, females of white-feather quails had higher villi lengths compared to males of the same variety. For crypt depth, females of the two quail varieties had deeper crypts compared to males. But the white-feathered females showed the deepest crypts among all. Similarly, for the villi width, the widest villi were found in females of the two varieties compared to males.

## Discussion

Based on the documented recessive plumage color mutations in Japanese quails (Petek et al., 2004), brown-, wild-, and white-feathered quail varieties are well defined (Bagh et al., 2016; ELSaidy et al., 2021). There are different explanations for how plumage color may affect different traits in quails, among these explanations is pleiotropic gene effect as pigmentation is controlled by genes that regulate the production of melanin, and carotenoids. Some of these genes have also been shown to be involved in the regulation of muscle development and growth in other species, suggesting that there may be a link between plumage color and carcass traits in quails. Several studies listed the physiological differences among these varieties focusing on the differences in body weights, growth rate, and other growth-related parameters (Maiorano et al., 2009; Tavaniello et al., 2014; Nasr et al., 2017). In this context, compared to the brown-feathered quails, the white-feathered quails displayed significant superiority and popularity on quail farms (Minvielle et al., 1999). However, the information about the fat deposition and carcass properties, during the growing period and with the progress of age among different quail populations is insufficient. Therefore, the current study represents, to the best of our knowledge, the first report about the differences in total body fat contents, carcass traits, and intestinal morphometry along with the growth performance among two different plumage color quail varieties, brown and white-feathered quails. Additionally, the study addressed the effects of variety by sex interaction on the above-mentioned characteristics. The key results of this study indicated significant variations in body weights, growth rate, and feed intake between the two quail varieties with the superiority of white-feathered quails over brown ones. These findings come in agreement with the results of the previous studies which stated the significant superiority of the white-feathered quails over the brown-feathered ones in terms of growth performance (Bagh et al., 2016; Nasr et al., 2017; Al-Kafajy et al., 2018). While some other studies reported contradicting findings, where white-feathered quails had lighter body weights than brown-feathered birds (Tarhyela et al., 2012; Mahmoud et al., 2014). These variations in the body have been shown to differ at different ages with the white-feathered quails having marked heavier body weight than the brown-feathered ones at 14 and 28 days of age (Jassim et al., 2006). These results may be explained by the significant associations of the feather color with growth performance (Inci et al., 2015; Al-Kafajy et al., 2018). Besides, these observations might be correlated with the differences in the intestinal morphometric parameters that displayed increasing in intestinal villi length and width in white compared to brown quails. Increasing intestinal villi length is linked with increasing nutrient absorption from the intestine. Thus, further future research is suggested to assess the absorption capacity differences among different quail populations by examining the transcriptomic levels of some nutrients absorption regulating genes. However, the contradictory findings of body weights in the present study and the previous ones among the different feathered-color quails are probably owing to the differences in the genetic structure, as well as environmental and management conditions (Vali, 2008)

When comparing the carcass yields, interesting variations in carcass weights and the internal organs weights especially gizzard and intestine along with weights of the total body fat were found between the two quail varieties and sex. These findings are comparable with that previously reported (Kaye et al., 2016; Nasr et al., 2017), which indicated that the white-feathered quails have higher carcass yields than the brown-feathered ones. The significant differences in total body fat which increased with increasing age among the two quail varieties can perhaps be correlated with the highest blood lipid constituents in white-feathered compared to the brown-feathered quails and decreasing body’s moisture content with the advancement of bird’s age (Abou-Kassem et al., 2019). Moreover, these differences in the carcass yields among different plumage-colored quails might be explained by the variations in the live body weights which affect the carcass and internal organs’ weights (Bacon and Nestor, 1975; Nasr et al., 2017). Besides, the superiority of the white-feathered quails over the brown-feathered ones in terms of carcass yields might indicate their eminent efficiency and the ability for better meat production (Nasr et al., 2017). Moreover, the sex by variety interaction effects on live weight, weight after slaughtering, weight after defeathering, and carcass weight were fluctuated, as it was significant at the 4^th^ week of age, and became insignificant at the 5^th^ and 6^th^ weeks of age. These fluctuations are in agreement with that previously reported in quails (Hassan and Abd–Alsattar 2016) and chickens (El-Henfnawy et al. 2022).

Regarding the meat quality properties, the marked high values of meat tenderness observed in both sex of brown-feathered quails compared to white ones might be attributed to the presence of dark muscle fibers with a smaller diameter confirmed by the higher color scores in those birds. Where, the tenderness of the meat is a multifactorial trait depending on numerous factors such as the percentage of tissue building up the muscle (Riegel et al., 2003; Reddy et al., 2017). Besides, water holding capacity exhibited a similar response with the brown feathered quails, which had higher values than white quails. This finding agreed with previous studies which reported that the differences in water-holding capacity are mainly attributed to variations in meat pH values (Van Laack et al., 2000; Lonergan et al., 2003). Meat color variations reported in this study are comparable to the results of (Abou-Kassem et al., 2019), which indicated variations in meat color are based on sex, where males had higher scores than females. Additionally, the reported variations in breast and thigh muscle weights might be correlated with the variations in live body weights among different quail varieties and sex.

The biochemical constituents showed significant variations among the two studied quail varieties in the level of albumen, and between males and females in globulin, Ca level and phosphorus contents as well as cholesterol and triglycerides levels. The differences in lipid parameters might be associated with the higher level of estrogen secretion in females compared to males which enhances the hepatic biosynthesis of triglycerides, phospholipids, lipoproteins, and cholesterol (Walzem et al., 1999). Moreover, the variations in total protein, albumen, and globulin agreed with Walzem et al results (Walzem et al., 1999), which attributed it to increasing lipoprotein synthesis with the onset of egg production and increasing steroid secretion. The findings of calcium and phosphorus levels agreed with (Pavlík et al., 2009; Abou-Kassem et al., 2019) findings, which displayed significant differences in Ca and phosphorus levels between males and females birds which might be returned to the increase in steroid hormones secretion which involved in Ca metabolism. In general, the reason for the observed variations among the studied quail varieties could be attributed to the quail’s population, feather color, and the method of estimation that may cause, to some extent, deviation from the observed results (Falconer and Mackay, 1983; Al-Kafajy et al., 2018).

Regarding egg quality properties, the obtained findings revealed a significant role of plumage color and quail varieties in controlling egg quality. White-feathered quails were superior to brown-feathered ones in shell thickness, and the percentage of shell and egg white. These findings agree with previous results that indicated significant variations in egg production among different quail lines (Ashok and Reddy, 2010; Al-Kafajy et al., 2018). Furthermore, (Yilmaz and Caglayan, 2008) confirmed the superiority of white-feathered quails in egg production in terms of egg weight. Nevertheless, other studies ascertained the superiority of the brown-feathered quails in egg production where they produced heavier eggs while the black quails produced more eggs than other plumage-colored quails (Ashok and Reddy, 2010). Furthermore, a prominent negative correlation between body weight and egg production has been ascertained (Silva et al., 2013; Baylan, 2017). The current findings indicate a significant correlation between plumage color and egg characteristics in quails. Though, determining the best quail variety in terms of egg production is quite controversial because there is no feasible and easy explanation for these variances among quail varieties because of the variation in the environmental conditions, and sampling error due to limited sample size (Al-Kafajy et al., 2018). For egg quality characteristics, most of the assessed parameters exhibited no significant differences. These results were comparable to those obtained by previous studies (Yilmaz and Caglayan, 2008; Al-Kafajy et al., 2018), which indicated no differences among different quail varieties. However, the distinct differences in eggshell thickness and percentage and albumen percentage between the white and brown quails disagreed with (Al-Kafajy et al., 2018), who stated the superiority of brown-feathered quails over the white ones in terms of egg quality (Al-Kafajy et al., 2018). This observation is perhaps explained by the differences in calcium and phosphorus levels between white and brown quails. Besides, it might reveal that the white-feathered quails possess better egg quality compared to the brown-feathered quails. As the albumen proprieties are crucial traits and feasible to improve egg quality, eggs with better albumen characters be likely to have better internal egg quality (Khawaja *et al.*, 2013).

## Conclusion

In light of the obtained findings, it can be concluded that white-feathered birds had, in general, higher values of productivity compared with brown-feathered quails during growing and laying periods. This is stated from the results of the growth performance including live body weights from hatch to 6-wk of age, weekly growth rate, feed intake, and FCR which indicated the superior performance of white-feathered quails over the brown-feathered ones. Besides, the white-feathered quails exceeded the brown ones in terms of carcass characteristics with the females were more superior compared to males plus a characteristic increase of total body fat with the proceeding of bird’s age. Remarkable variations between the studied quail varieties, with significant dominance of females in both varieties, at the level of water holding capacity, pH, and meat tenderness ascertained an obvious superiority of white-feathered quails compared to brown ones and indicated the higher tendency of the white quails for meat production. These results were linked with significant changes in biochemical profiles including lipids biomarkers, total protein, and Ca and phosphorus levels along with variations in the intestinal morphometry. Despite the higher growth performance of white quails, they recorded less egg production (%) compared to brown ones. However, white quails possessed better egg quality properties such as an increased egg-shell thickness and % along with albumin % compared to brown quails.

It is worth to mention that the exact mechanisms by which plumage color affects different traits in quails are not fully understood, Therefore, more research is needed to fully elucidate these relationships and to determine the genetic and physiological factors that underlie them.

## Data Availability

Data available upon request from the authors.
